# *Botrytis cinerea* tolerates phytoalexins produced by Solanaceae and Fabaceae plants through an efflux transporter BcatrB and metabolizing enzymes

**DOI:** 10.3389/fpls.2023.1177060

**Published:** 2023-06-02

**Authors:** Abriel Salaria Bulasag, Maurizio Camagna, Teruhiko Kuroyanagi, Akira Ashida, Kento Ito, Aiko Tanaka, Ikuo Sato, Sotaro Chiba, Makoto Ojika, Daigo Takemoto

**Affiliations:** ^1^ Graduate School of Bioagricultural Sciences, Nagoya University, Nagoya, Japan; ^2^ College of Arts and Sciences, University of the Philippines Los Baños, Los Baños, Laguna, Philippines

**Keywords:** ABC transporter, *Botrytis cinerea*, detoxification, phytoalexins, plant disease resistance

## Abstract

*Botrytis cinerea*, a plant pathogenic fungus with a wide host range, has reduced sensitivity to fungicides as well as phytoalexins, threatening cultivation of economically important fruits and vegetable crops worldwide. *B. cinerea* tolerates a wide array of phytoalexins, through efflux and/or enzymatic detoxification. Previously, we provided evidence that a distinctive set of genes were induced in *B. cinerea* when treated with different phytoalexins such as rishitin (produced by tomato and potato), capsidiol (tobacco and bell pepper) and resveratrol (grape and blueberry). In this study, we focused on the functional analyses of *B. cinerea* genes implicated in rishitin tolerance. LC/MS profiling revealed that *B. cinerea* can metabolize/detoxify rishitin into at least 4 oxidized forms. Heterologous expression of Bcin08g04910 and Bcin16g01490, two *B. cinerea* oxidoreductases upregulated by rishitin, in a plant symbiotic fungus *Epichloë festucae* revealed that these rishitin-induced enzymes are involved in the oxidation of rishitin. Expression of *BcatrB*, encoding an exporter of structurally unrelated phytoalexins and fungicides, was significantly upregulated by rishitin but not by capsidiol and was thus expected to be involved in the rishitin tolerance. Conidia of *BcatrB* KO (*ΔbcatrB*) showed enhanced sensitivity to rishitin, but not to capsidiol, despite their structural similarity. *ΔbcatrB* showed reduced virulence on tomato, but maintained full virulence on bell pepper, indicating that *B. cinerea* activates *BcatrB* by recognizing appropriate phytoalexins to utilize it in tolerance. Surveying 26 plant species across 13 families revealed that the *BcatrB* promoter is mainly activated during the infection of *B. cinerea* in plants belonging to the Solanaceae, Fabaceae and Brassicaceae. The *BcatrB* promoter was also activated by *in vitro* treatments of phytoalexins produced by members of these plant families, namely rishitin (Solanaceae), medicarpin and glyceollin (Fabaceae), as well as camalexin and brassinin (Brassicaceae). Consistently, *ΔbcatrB* showed reduced virulence on red clover, which produces medicarpin. These results suggest that *B. cinerea* distinguishes phytoalexins and induces differential expression of appropriate genes during the infection. Likewise, BcatrB plays a critical role in the strategy employed by *B. cinerea* to bypass the plant innate immune responses in a wide variety of important crops belonging to the Solanaceae, Brassicaceae and Fabaceae.

## Introduction

*Botrytis cinerea*, commonly known as grey mold, is one of the most economically important pathogens. It affects approximately 1,400 plant species across several plant families (Elad et al., 2016) and brings severe yield losses to high impact crops such as grapevine, Solanaceae (tomato, potato, bell pepper), Brassicaceae (canola, cabbage, broccoli), Fabaceae (pea, bean, soybean) among others. However, recent studies employing multi-omic strategies hint that *B. cinerea’*s potency as a pathogen as well as the evolutionary path towards broad host necrotrophy remains largely an enigma (Hahn et al., 2014; Frías et al., 2016; Derbyshire et al., 2017; Valero-Jiménez et al., 2019). Similarly, virulence across plant species is enabled by complex interactions that include key fungal processes such as oxidative stress response (N'Guyen et al., 2021) and cell wall integrity (Cui et al., 2021; Escobar-Niño et al., 2021).

*B. cinerea* craftily manipulates plant defenses, resulting in necrosis of host tissues by means of plant cell death inducing proteins (PCIDs) (Petrasch et al., 2019; Leisen et al., 2022), creating a favorable environment for necrotrophy. Thereafter, it prompts the plant host to an exchange of enzymes and toxins, modulated by phytohormone crosstalk, to launch an induced systemic resistance (Mbengue et al., 2016; Bi et al., 2022). An important plant defense mechanism against necrotrophic pathogens is the production of phytoalexins, a diverse group of compounds which are toxic to invading organisms. These phytoalexins were shaped and diversified through phylogenetic lineages and plant-pathogen interaction events, which led to the formation of a wide variety of structurally distinct compounds. Structural variations, impeccable timing and synergistic action among these phytoalexins offer an effective counter to most fungal pathogens (Pedras and Abdoli, 2017; Allan et al., 2019; Newman and Derbyshire, 2020; N'Guyen et al., 2021; Westrick et al., 2021; Zhou et al., 2022). In some cases, phytoalexin-mediated autophagy also drove non-host resistance against necrotrophs (Sanchez-Vallet et al., 2010; Prasad et al., 2022). Hence, detoxification of phytoalexins became imperative and paved the success of cosmopolitan phytopathogens such as *Sclerotinia* and *Botrytis* (Westrick et al., 2019; Kusch et al., 2022). In concrete terms, robust fungal pathogenic systems deploy cytochrome P450 genes, multi-functional enzymes that confer virulence through nullification of diverse phytotoxins (van den Brink et al., 1998; Pedras and Ahiahonu, 2005). Their versatility enables numerous ways to metabolize phytoalexins, of which oxidoreduction takes pre-eminence (Shin et al., 2018; Kuroyanagi et al., 2022). Despite having extensive descriptions of phytoalexin-detoxification products, few cytochrome P450 genes have been functionally characterized in fungi as compared to plant and animal systems (Park et al., 2008; Chen et al., 2014).

Non-degradative detoxification mechanisms play an equally significant role in tolerating phytoalexins, especially among broad-host range pathogens such as *B. cinerea*. Membrane-bound fungal transporters were deemed to be key not only in regulating endogenous phytotoxins but also xenobiotics such as fungicides which further augments flexibility among generalist pathogens (Pedras et al., 2011; Hahn et al., 2014; Vela-Corcía et al., 2019; Chen et al., 2020). Using combined transcriptomic and metabolomic approaches, multidrug resistance to fungicides was found to be primarily aided by efflux transporter genes enhancing virulence of key fungal pathogens (Kretschmer et al., 2009; Zhang et al., 2019; Samaras et al., 2020; Shi et al., 2020; Wang et al., 2021; Bartholomew et al., 2022; Harper et al., 2022; Wang et al., 2022). More specifically, ABC (ATP-binding cassette) and MFS (major facilitator superfamily) transporters dispose key phytoalexins by exporting them out of fungal cells (Newman and Derbyshire, 2020; Kumari et al., 2021; Madloo et al., 2021; Westrick et al., 2021) paving the way to generalist pathogen lineages. Functional analysis of transporter genes such as *BcatrB* and *mfsM2*, elucidated the contribution of these genes to multidrug and fungicide resistance (Kretschmer et al., 2009). This provides crucial insight into the control of economically important fungal pathogens such as *B. cinerea*. Fungal transporters, encoded by single genes, can be conveniently transferred across fungal phyla through horizontal gene transfer, leading to enhanced polyxenous behavior and plant host colonization (Soanes and Richards, 2014; Milner et al., 2019).

In summary, the mechanism conferring generalist pathogens to overcome phytoalexins and drastically expand their host range is likely driven by two important processes: enzymatic transformation of key antimicrobial metabolites; and efflux processes facilitated by membrane transporters. Hence, this study aspires to provide a novel perspective in understanding molecular mechanisms governing the interaction between phytoalexin production in various plant model systems and its subsequent metabolism and efflux in the polyxenous grey mold pathogen *B. cinerea*.

## Materials and methods

### Biological material, growth conditions and incubation in phytoalexins

*Botrytis cinerea* strain AI18 (Kuroyanagi et al., 2022), *Epichloë festucae* strain Fl1 (Young et al., 2005) and their transformants used in this study are listed in **Supplementary Table 1**. They were grown on potato dextrose agar (PDA) at 23°C. For the incubation in phytoalexins, mycelia plugs (approx.1 mm^3^) were excised from the growing edge of the colony using a dissection microscope (Stemi DV4 Stereo Microscope, Carl Zeiss, Oberkochen, Germany) and submerged in 50 μl of water or indicated phytoalexin in a sealed 96 well clear plate. The plate was incubated at 23°C for the indicated time.

Capsidiol was purified from *Nicotiana tabacum* as previously reported (Matsukawa et al., 2013) and synthesized rishitin (Murai et al., 1975) was provided by former Prof. Akira Murai (Hokkaido University, Japan). Resveratrol and brassinin were obtained from Sigma-Aldrich (Burlington, MA, USA). Glyceollin (glyceollin I) was obtained from Wako pure chemical (Osaka, Japan). Medicarpin was obtained from MedChemExpress (Monmouth Junction, NJ, USA).

### Detection of rishitin and their metabolites using LC/MS

For the detection of rishitin and their metabolites after the incubation with *B. cinerea* or *E. festucae* transformants, the supernatant (50 μl) was collected, mixed with 50 μl acetonitrile and measured by LC/MS (Accurate-Mass Q-TOF LC/MS 6520, Agilent Technologies, Santa Clara, CA, USA) with ODS column Cadenza CD- C18, 75 x 2 mm (Imtakt, Kyoto, Japan) as previously described (Kuroyanagi et al., 2022).

### RNAseq analysis

Extraction of RNA and RNA sequencing analysis were performed as previously described (Rin et al., 2020; Kuroyanagi et al., 2022). The nucleotides of each read with less than 13 quality value were masked and reads shorter than 50 bp in length were discarded, and filtered reads were mapped to annotated cDNA sequences for *B. cinerea* (ASM83294v1, GenBank accession GCA_000143535) using Bowtie software (Langmead et al., 2009). For each gene, the relative fragments per kilobase of transcript per million mapped reads (FPKM) values were calculated and significant difference from the control was assessed by the two-tailed Student’s *t*-test. RNA-seq data reported in this work are available in GenBank under the accession numbers DRA013980.

### Extraction of genomic DNA, PCR and construction of vectors

Genomic DNA of *B. cinerea* was isolated from fungal mycelium grown in potato dextrose broth (PDB) using DNeasy Plant Mini Kit (QIAGEN, Hilden, Germany). PCR amplification from genomic and plasmid DNA templates was performed using PrimeStar Max DNA polymerase (Takara Bio, Kusatsu, Japan) or GoTaq Master Mix (Promega, Madison, WI, USA). Vectors for heterologous expression, detection of promoter activity, gene knock out used in this study are listed in **Supplementary Table 2** (Tanaka et al., 2008; Niones and Takemoto, 2015). For the heterologous expression of *B. cinerea* Bcin08g04910 and Bcin16g01490 genes in *E. festucae*, *Aureobasidium pullulans* TEF promoter (Vanden Wymelenberg et al., 1997) was used for constitutive expression (Takemoto et al., 2006). For the production of reporter strains, codon-optimized GFP for *B. cinerea* (Leroch et al., 2011) or codon-optimized *Luc* gene for *Neurospora crassa* (Gooch et al., 2008) was used as previously reported (Kuroyanagi et al., 2022). Sequences of primers used for the construction of vectors and PCR to confirm the gene knockout are listed in **Supplementary Table 3**.

### Fungal transformation

Protoplasts of *E. festucae* and *B. cinerea* were prepared from freshly grown mycelia or geminating conidia, respectively, as previously described (Kuroyanagi et al., 2022). Protoplasts were transformed with 5 µg of either circular or linear (for gene knock out) plasmids using the method previously described (Kuroyanagi et al., 2022). Candidate colonies were exposed to BLB blacklight for the induction of sporulation and single spore isolation was performed to obtain purified strains. Note that *ΔbcatrB*-14 and -23 were isolated from separate transformation experiments. Transformants of *E. festucae* and *B. cinerea* used in this study are listed in **Supplementary Table 1** (Kayano et al., 2013).

### Pathogen inoculation

Leaves or fruits (tomato, grape) of plant species were kept moistened and sealed in a plastic chamber. Leaves detached from the plant were covered with a wet tissue at the cut end of the stem. Mycelial plugs (approx. 5 mm x 5 mm) of *B. cinerea* were excised from the growing edge of the colony grown on PDA and placed on the abaxial side of the leaf or on the fruit and covered with wet lens paper. For the inoculation on tomato, mycelial blocks of *B. cinerea* were placed on the cut surface of tomato, and the fruits were kept at high humidity at 23°C for 5 days. *B. cinerea* conidia formed on tomato were washed off in 15 ml water, and number of conidia in water was counted using a hemocytometer.

### Microscopy

Images of *B. cinerea* expressing GFP under the control of the *BcatrB* promoter were collected using a confocal laser scanning microscope FV1000-D (Olympus, Tokyo, Japan). The laser for detection of GFP was used as the excitation source at 488 nm, and GFP fluorescence was recorded between 515 and 545 nm. Images were acquired with settings that did not saturate the fluorescence, and the total fluorescence per spore was determined using ImageJ software (Schneider et al., 2012).

### Detection of luciferase activity in *B. cinerea* P_*BcatrB* : *Luc* transformant

*B. cinerea* P_*BcatrB* : Luc transformant was grown on PDA at 23°C. Three mycelia plugs (approx. 2 mm x 2 mm) were excised from the growing edge of the colony and submerged in 50 μl of water or indicated phytoalexin containing 50 μM D-luciferin in a sealed 96-well microplate (Nunc 96F microwell white polystyrene plate, Thermo Fisher Scientific, Waltham, MA, USA). Changes in luminescence intensity were measured over time with Mithras LB 940 (Berthold Technologies, Bad Wildbad, Germany).

## Results

### Rishitin treatment induced genes predicted to be involved in the metabolization and efflux of the phytoalexin in *B. cinerea*

Previously, we have performed RNA-seq analysis of *B. cinerea* in response to sesquiterpenoid (rishitin and capsidiol) and stilbenoid (resveratrol) phytoalexins. *Bccpdh*, encoding a short chain dehydrogenase, was identified as a gene specifically induced in *B. cinerea* treated with capsidiol, and BcCPDH was revealed to be involved in the detoxification of capsidiol to less toxic capsenone (Kuroyanagi et al., 2022).

**Figure 1:**
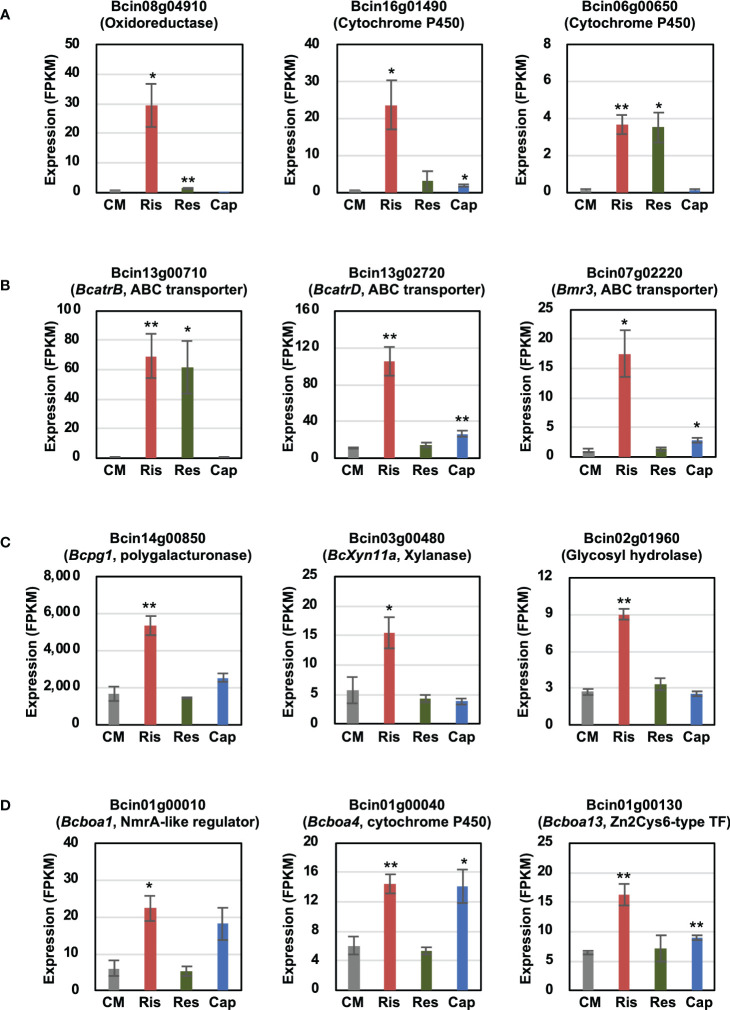
Transcriptional changes in *Botrytis cinerea* genes induced by rishitin. **(A)** Cytochrome P450/oxidoreductases, **(B)** ABC transporters, **(C)** Cell wall degrading enzymes (CWDEs) and **(D)** Botcinic acid biosynthesis genes. TF, transcription factor. The gene expression (FPKM value) was determined by RNA-seq analysis of *B. cinerea* cultured in CM media containing 500 µM rishitin, 500 µM resveratrol or 100 µM capsidiol for 24 h. Data are mean ± SE (*n* = 3). Asterisks indicate a significant difference from the control (CM) as assessed by two-tailed Student’s *t*-test, **P < 0.01, *P < 0.05.

In this study, we focused on genes induced by rishitin treatment. To identify rishitin-induced genes, we profiled the transcriptome of rishitin-treated *B. cinerea* using RNAseq analysis. Differentially expressed genes included genes from various gene families in *B. cinerea*, such as cytochrome P450/oxidoreductases, ABC transporters, cell wall degrading enzymes (CWDEs), and secondary metabolite synthesis (**Figure 1**). Expression of several ABC transporter genes was also induced and may potentially be involved in the efflux of rishitin to enhance the tolerance of *B. cinerea* (**Figure 1B**). Our RNAseq data indicates that expression of *BcatrB* (Bcin13g00710) is induced in rishitin and resveratrol, but not in capsidiol. Conversely, expression of *BcatrD* (Bcin13g02720) and *Bmr3* (Bcin07g02220) was significantly higher during rishitin treatment as compared to other phytoalexins. While the ABC transporter genes described above are induced in rishitin-treated *B. cinerea*, expression of a different set of transporter genes is activated when treated with capsidiol (Kuroyanagi et al., 2022). Interestingly, some genes that are not directly involved in the detoxification of phytoalexins, but instead related to the virulence of *B. cinerea*, are upregulated in *B. cinerea* treated with rishitin. For example, expression of genes encoding CWDEs, such as genes for polygalacturonase *Bcpg1* (Bcin14g00850), xylanase *BcXyn11a* (Bcin02g01960) and glycosyl hydrolase (Bcin02g01960), were induced by rishitin treatment (**Figure 1C**). Genes that are part of a subtelomeric cluster for the production of phytotoxic botcinic acid are also activated by rishitin and capsidiol treatment (**Figure 1D**). Further, some genes induced by rishitin were potentially involved in the metabolization of rishitin into its oxidized form, including an oxidoreductase (Bcin08g04910) and cytochrome P450 genes (Bcin16g01490, Bcin06g00650, Bcin07g05430) (**Figure 1A**, Kuroyanagi et al., 2022). As such these genes were investigated further in terms of their ability to metabolize rishitin.

### Heterologous expression of rishitin-induced *B. cinerea* genes in symbiotic fungus results in the metabolization of rishitin to its oxidized forms

**Figure 2:**
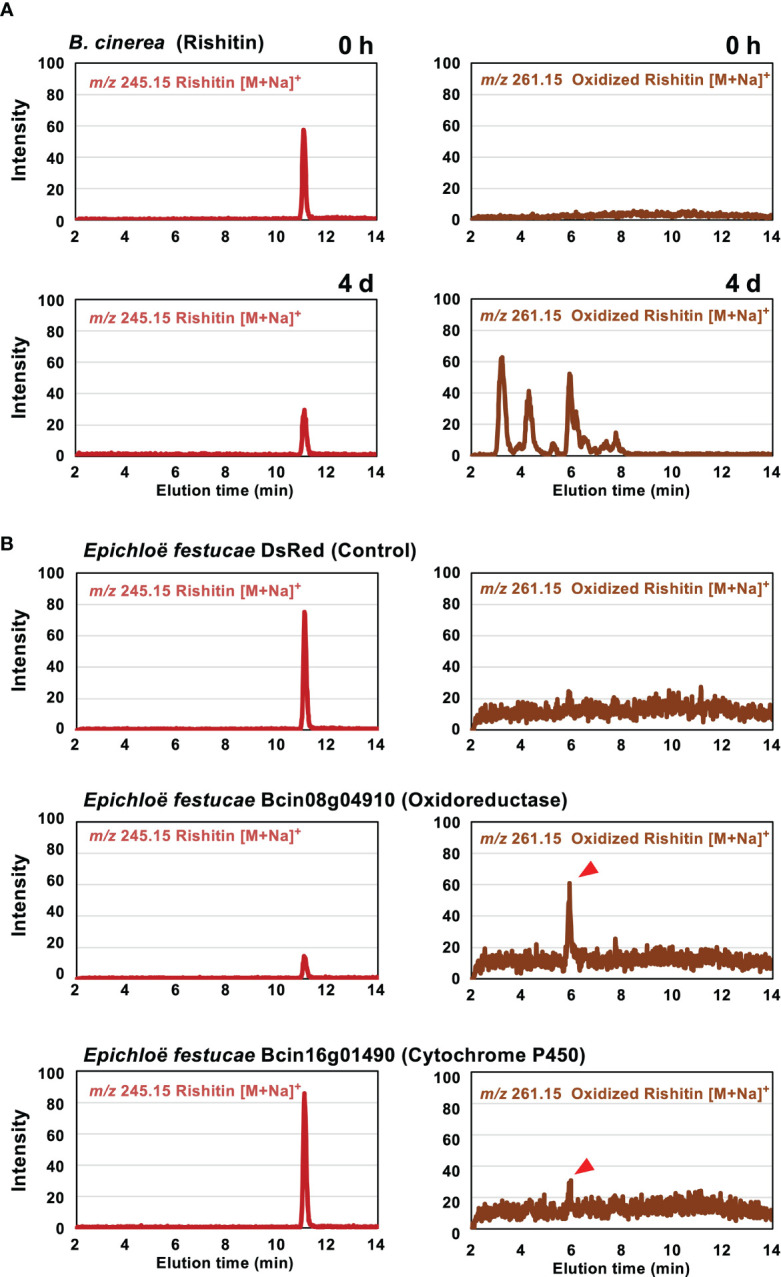
Metabolization of rishitin by *Botrytis cinerea* and *Epichloë festucae* transformants expressing rishitin-induced *B. cinerea* genes. **(A)** Mycelial block (approx. 1 mm^3^) of *B. cinerea* was incubated in 50 µl of 500 µM rishitin for 4 days and remaining rishitin and oxidized rishitin were detected by LC/MS. **(B)** Mycelial block (approx. 1 mm^3^) of *E festucae* transformants expressing *DsRed* gene (control) or rishitin-induced *B. cinerea* genes (Bcin08g04910 or Bcin16g01490) were incubated in 50 µl of 100 µM rishitin for 2 days and remaining rishitin and oxidized rishitin were detected by LC/MS. See **Supplementary Figure 1** for 10 days incubation of *E festucae* transformants expressing Bcin16g01490 in rishitin.

Rishitin is metabolized to at least 4 oxidized forms by *B. cinerea* (**Figure 2A**, Kuroyanagi et al., 2022). In potato, which produces rishitin, oxidation of rishitin is known as a detoxification reaction (Camagna et al., 2020). To investigate the function of rishitin-induced *B. cinerea* cytochrome P450 and oxidoreductase genes in rishitin oxidation (**Figure 1A**), candidate genes were heterologously expressed in the grass symbiotic fungus *Epichloë festucae* to detect the enzymatic activity of the encoded proteins. Kuroyanagi et al. (2022) have used the same system to identify a dehydrogenase BcCPDH, which can convert capsidiol to capsenone. Two genes upregulated in *B. cinerea* treated with rishitin, Bc08g04910 and Bc16g01490, were expressed in *E. festucae* under the control of the TEF promoter (Vanden Wymelenberg et al., 1997) for constitutive expression. These *E. festucae* transformants were incubated in rishitin solution to examine their function in rishitin oxidation. Two days after incubation in 100 µM rishitin, the *E. festucae* strain expressing Bcin08g04910 caused a reduction of rishitin, whereupon oxidized rishitin was detected (**Figure 2B**). Similarly, oxidized rishitin was detected after the incubation of rishitin with the *E. festucae* strain expressing Bcin16g01490, although a significant reduction of rishitin was not observed after 2 days. Reduction of rishitin and pronounced production of two oxidized rishitin derivatives was observed after 10 days of incubation with *E. festucae* expressing Bcin16g01490 (**Supplementary Figure 1**). *E. festucae* control strains expressing *DsRed* did not induce a reduction or oxidation of rishitin (**Figure 2B**). While *B. cinerea* produced various rishitin metabolites after the incubation with rishitin, each of the *E. festucae* transformants showed one or two peaks, which demonstrated an increase in mass, indicative of the presence of an additional oxygen atom in the rishitin molecule. These results suggest that enzymes encoded by Bc08g04910 and Bc16g01490 can metabolize rishitin into oxidized forms. Employing these two *E. festucae* transformants in large scale incubation with rishitin should enable further insights into the chemical structure of the oxidized rishitin compounds. This study has yet to identify all genes that correspond to the metabolization of rishitin into several oxidized forms by the wild-type *B. cinerea* strain. Multiple genes are involved in the detoxification/oxidation of rishitin (**Figure 2B**), KO of single gene may therefore not have a significant effect on the virulence of this pathogen.

### *BcatrB* KO mutants showed increased sensitivity to rishitin

**Figure 3:**
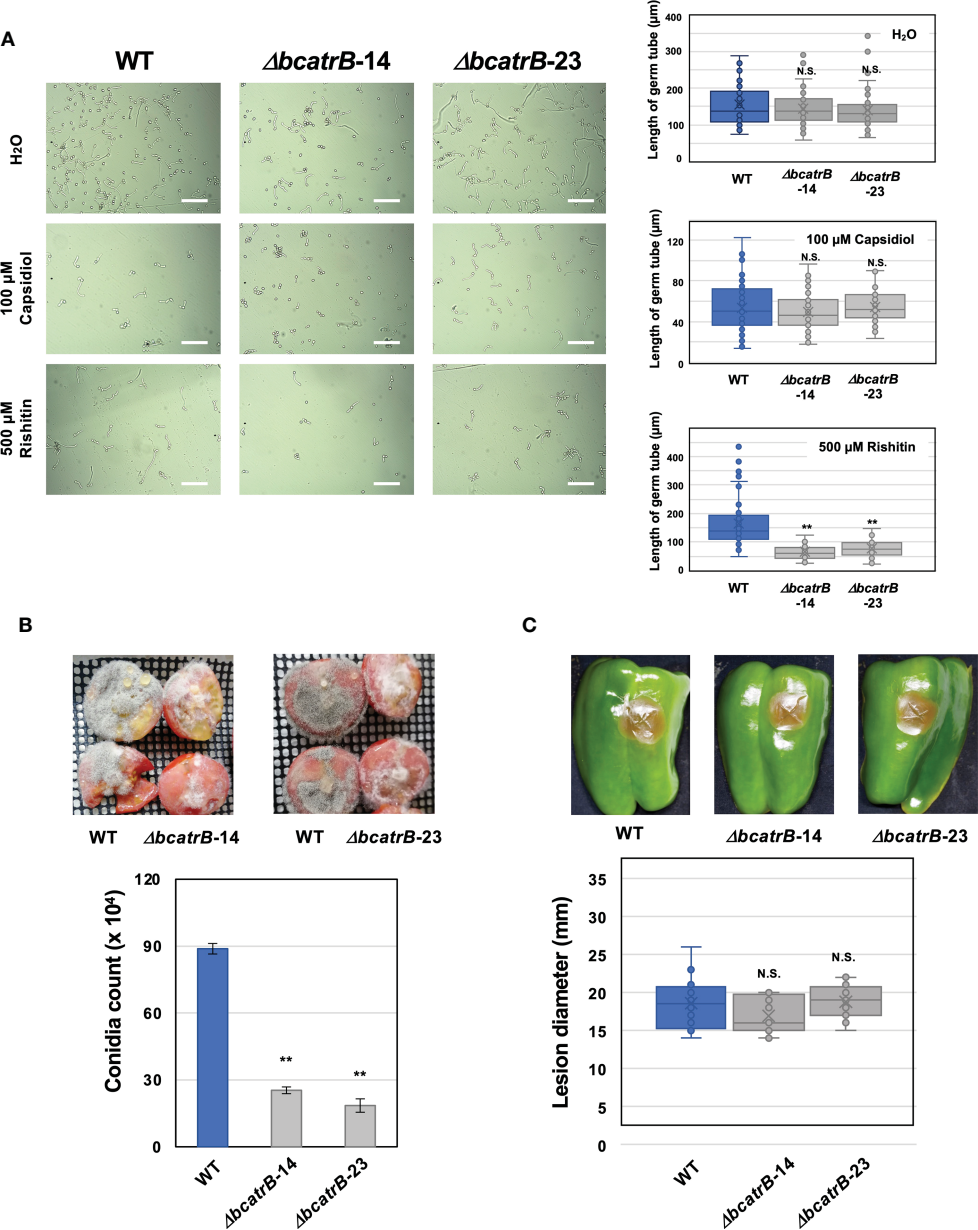
Deletion of *BcatrB* gene compromises the rishitin tolerance of *Botrytis cinerea.* **(A)** Conidial suspension of *B. cinerea* was incubated in water (H_2_O), 100 µM capsidiol or 500 µM rishitin and the length of germ tube was measured after 18 h incubation. Bars = 100 µm. Data are mean ± SE (n = 60). Asterisks indicate a significant difference from WT as assessed by two-tailed Student’s *t*-test. **P < 0.01.* N*. S., not significant. Lines and crosses (x) in the columns indicate the median and mean values, respectively. **(B)** Tomato fruits (cut in half) were inoculated with mycelia plug (approx. 5 x 5 mm) of *B. cinerea* wild type (WT) or *ΔbcatrB* strains and produced conidia were counted 7 days after the inoculation. Data are mean ± SE (n = 3). Asterisks indicate a significant difference from WT as assessed by two-tailed Student’s *t*-test. **P < 0.01. **(C)** Fruits of bell pepper (*Capscicum annuum*) were inoculated with mycelia plug (approx. 5 mm diameter) of *B. cinerea* WT or *ΔbcatrB* strains. Data are mean ± SE (n = 12). N. S. indicate no significant difference from WT as assessed by two-tailed Student’s *t*-test.

Previous studies on *BcatrB* established its role in the tolerance of *B. cinerea* to structurally unrelated phytoalexins such as resveratrol and camalexin, as well as to the fungicide fenpicionil (Schoonbeek et al., 2001; Vermeulen et al., 2001; Stefanato et al., 2009). Expression of *BcatrB* is upregulated by rishitin (**Figure 1B**), thus *BcatrB* is presumably involved in the tolerance of *B. cinerea* to rishitin. To investigate the role of *BcatrB* in rishitin tolerance of *B. cinerea*, *BcatrB* knock out strains (*ΔbcatrB*) were generated (**Supplementary Figure 2**). Conidial germination of wild type and *ΔbcatrB* strains were measured after treatment with rishitin or capsidiol (**Figure 3A**). In 100 µM capsidiol, the length of *B. cinerea* germ tubes were comparatively shorter than untreated conidia (**Figure 3A**). However, knock out of *BcatrB* yielded no significant effect on the sensitivity of *B. cinerea* to capsidiol, consistent with the RNAseq data that *BcatrB* is not induced under capsidiol treatment (**Figure 1B**). In contrast, *BcatrB* mutants showed enhanced sensitivity to rishitin compared with wild type strain (**Figure 3A**). Given that rishitin production has been detected in tomato fruits, but not in leaves, upon pathogen attack (Sato et al., 1968, de Wit and Flach, 1979), virulence of *BcatrB* strains was tested on tomato fruits. A higher sporulation rate was observed for the wild type compared to the *ΔbcatrB* strains in tomato fruits (**Figure 3B**), while the sporulation rate of the complemented strain was comparable to the wild type (**Supplementary Figure 3A**). In tomato leaves, the difference in the development of disease symptoms between wild and KO strains was not as pronounced as in fruit (**Supplementary Figure 4A**). Moreover, no significant differences were observed between wild type and *ΔbcatrB* strains in the lesion formation on bell pepper fruits (**Figure 3C**), which produce capsidiol as a major phytoalexin (Watson and Brooks, 1984). These results suggest that *BcatrB* is crucial in the tolerance of *B. cinerea* to rishitin, but not to capsidiol.

### *B. cinerea BcatrB* promoter is activated during the infection in plants belonging to Solanaceae, Brassicaceae and Fabaceae

**Figure 4:**
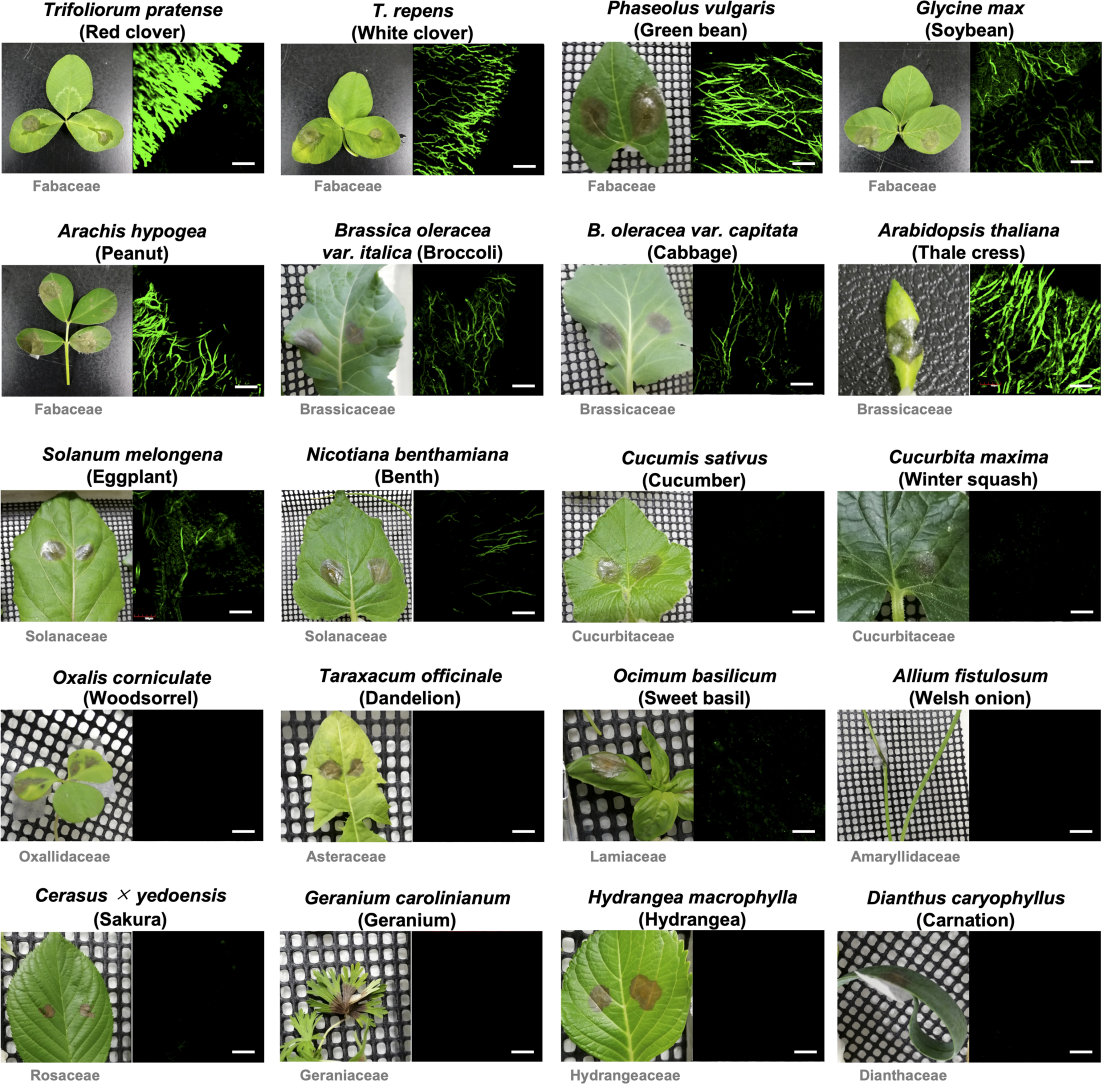
*Botrytis cinerea BcatrB* promoter is activated during the infection in Fabaceae, Brassicaceae and Solanaceae species. Leaves of indicated plants were inoculated with the mycelia of *B. cinerea* P_*BcatrB* : *GFP* transformant and hyphae at the edge of the lesion were observed by confocal laser microscopy 2 or 3 d after the inoculation. Bars = 100 µm.

**Figure 5:**
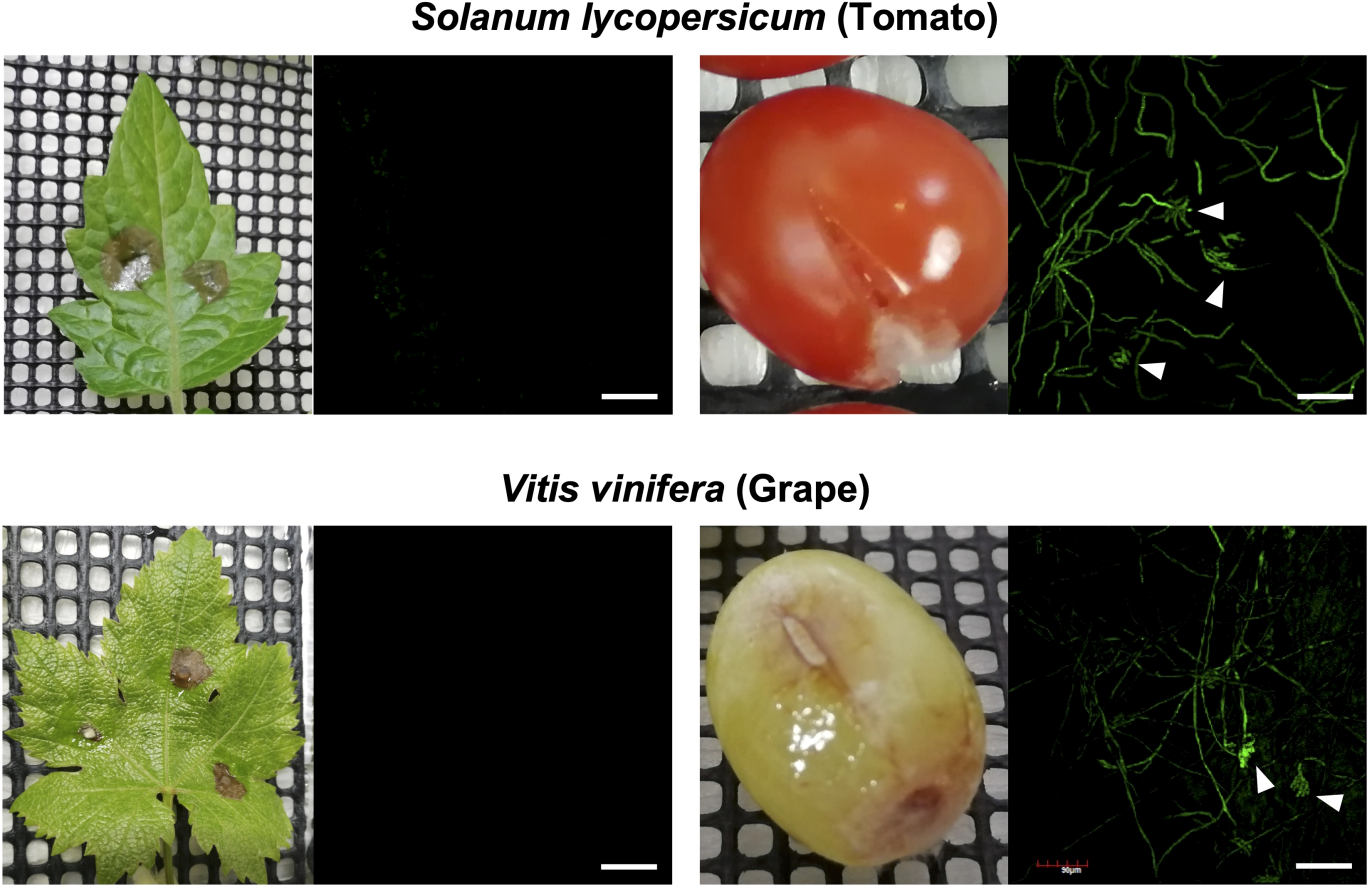
*Botrytis cinerea BcatrB* promoter is activated during the infection in fruits of tomato and grape. Leaves or fruits of tomato (top) or grape (bottom) were inoculated with the mycelia of *B. cinerea* P_*BcatrB* : *GFP* transformant and hyphae at the edge of the lesion was observed by confocal laser microscopy 2 or 3 d after the inoculation. Arrowheads indicate infection cushions. Bars = 100 µm.

In previous studies using GUS and GFP reporter strains of *B. cinerea*, it was shown that the expression of *BcatrB* is induced during the infection in Arabidopsis or upon treatment with camalexin, eugenol (Brassicaceae phytoalexins) or several fungicides (Stefanato et al., 2009; Leroch et al., 2011). To explore other phytoalexins that could be potential substrates of the BcatrB transporter, *B. cinerea* transformants expressing GFP under the control of the 1 kb *BcatrB* promoter (P_*BcatrB_GFP*) were inoculated onto 25 host plants across 13 plant families. Among the plant species surveyed, expression of GFP was mostly detected during the infection in Brassicaceae, Fabaceae and Solanaceae species (**Figure 4**, **Supplementary Table 4**). GFP expression ranged from weak, such as in the case of eggplant and *N. benthamiana*, to intense signals detected from red clover and Arabidopsis incubations. Similarly, activity of the *BcatrB* promoter was perceived to widely vary across members of the same family. The intensity of promoter activation also varied among plants at lower taxonomic levels such as in the case of red clover and white clover (genus *Trifolium*). Moreover, tissue-specific expression was also observed for tomato and grape, with *BcatrB* being strongly expressed in fruits but weak or no expression in leaves (**Figure 5**). *BcatrB* expression was also detected in infection cushions, which were only formed in fruit tissues (**Figure 5**).

### *BcatrB* is involved in virulence expression on red clover, a Fabaceae plant producing pterocarpan phytoalexins

**Figure 6:**
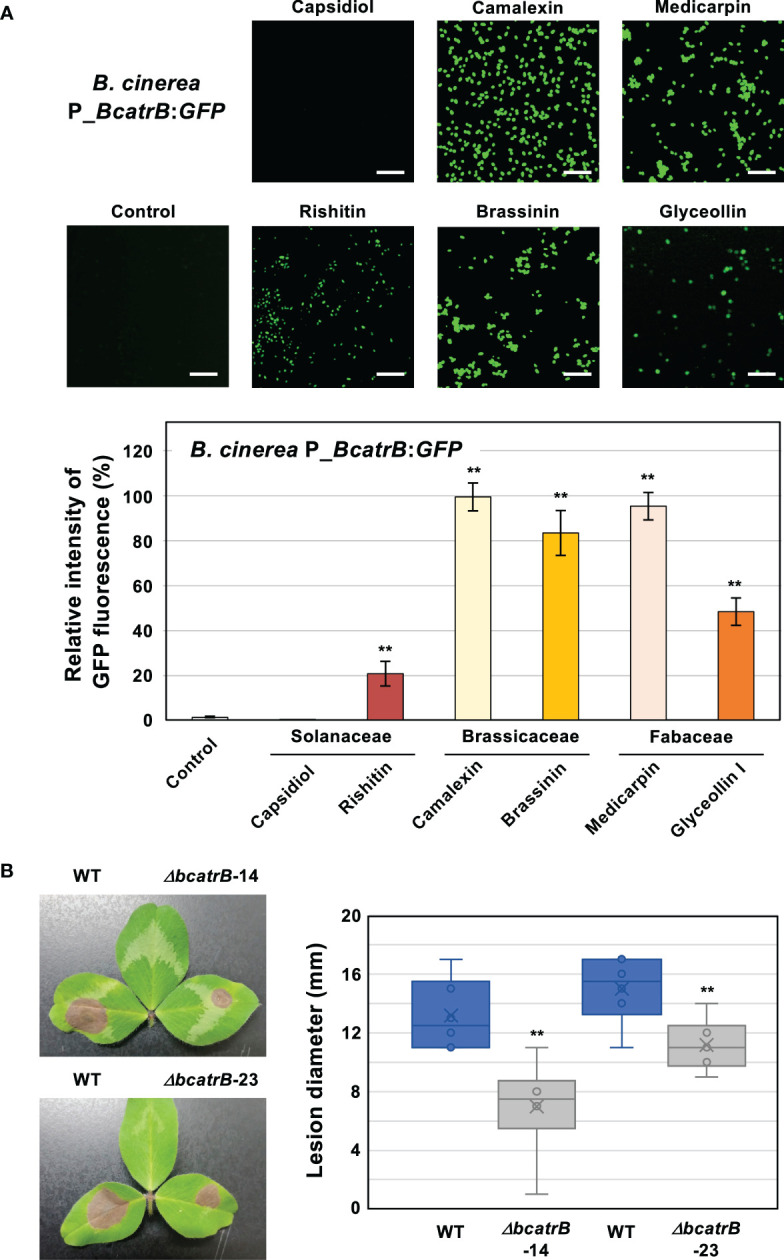
**(A)** *Botrytis cinerea BcatrB* promoter is activated by Solanaceae, Brassicaceae and Fabaceae phytoalexins. Conidia of *B. cinerea* P_*BcatrB* : *GFP* were treated with 1% DMSO (Control) or 100 µM of indicated phytoalexins, and GFP fluorescence was detected by confocal laser microscopy 2 days after the treatment. Bars = 100 µm. Data are mean ± SE (n = 20). Asterisks indicate a significant difference from control as assessed by two-tailed Student’s *t*-test. **P < 0.01. **(B)** Leaves of red clover (*Trifolium pratense*) were inoculated with mycelia plug (approx. 5 x 5 mm) of *B. cinerea* wild type (WT) or *ΔbcatrB* strains and lesion diameter was measured 3 days after the inoculation. Data are mean ± SE (n = 6). Asterisks indicate a significant difference from WT as assessed by two-tailed Student’s *t*-test. **P < 0.01.

To evaluate the effect of *in vitro* phytoalexin treatment on *BcatrB* activation, conidia of P_*BcatrB_GFP* transformant were treated with several phytoalexins from Brassicaceae, Fabaceae and Solanaceae species. Consistent with the RNAseq analysis data, the *BcatrB* promoter was activated by rishitin, but not by capsidiol (**Figure 6A**). The activation of the *BcatrB* promoter in *N. benthamiana* (**Figure 4**), which mainly produces capsidiol, is presumably due to its response to other antimicrobial substances produced in *N. benthamiana* (Shibata et al., 2016; Imano et al., 2022). Indole phytoalexins from Brassicaceae species, brassinin and camalexin, significantly activated the expression of GFP under the control of the *BcatrB* promoter*.* Pterocarpan phytoalexins produced in Fabaceae, medicarpin and glyceollin, also induced the activation of the *BcatrB* promoter (**Figure 6A**). Therefore, activation of the *BcatrB* promoter during the infection of Brassicaceae and Fabaceae plants (**Figure 4**) is linked to the recognition of indole and pterocarpan phytoalexins by *B. cinerea*.

*B. cinerea* during the infection of red clover (*Trifolium pratense*) exhibited the most substantial activation of the *BcatrB* promoter (**Figure 4**). To further substantiate *BcatrB* as a critical virulence factor for the infection of red clover, we performed inoculations using the *ΔbcatrB* KO strains. Compared to the wild type strain, the *ΔbcatrB* strains resulted in lower lesion scores (**Figure 6B**), indicating that *BcatrB* is required for full virulence on red clover, which produces the pterocapan phytoalexins medicarpin and maackiain (Dewick, 1975). Virulence was recovered upon complementation, with WT and *ΔbcatrB-14-C1* strains yielding similar lesion scores, confirmed the importance of *BcatrB* for the virulence of *B. cinerea* on red clover (**Supplementary Figure 3B**).

Leaves of green bean, broccoli and cabbage were also inoculated with *ΔbcatrB* KO strains. There was a trend showing a slight decrease in lesion size of KO strains compared with wild type (**Supplementary Figures 4B-D**), although the difference was not as apparent compared to red clover, where activation of *BcatrB* promoter was markedly more intense (**Figure 4**).

### *B. cinerea* rapidly activates the *BcatrB* promoter in response to Solanaceae, Brassicaceae and Fabaceae phytoalexins

**Figure 7:**
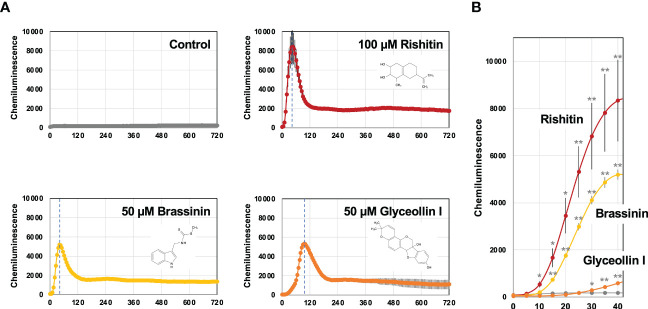
Activation of the *BcatrB* promoter detected in *Botrytis cinerea* transformant expressing *Luciferase* gene under the control of 2 kb *BcatrB* promoter (P_*BcatrB* : *Luc*). **(A)** P_*BcatrB* : *Luc* transformant was incubated in 1% DMSO (control), 100 µM rishitin, 50 µM brassinin or 50 µM glyceollin containing 50 µM D-Luciferin (substrate of luciferase). Data are mean ± SE (*n* = 3). Dotted vertical lines indicate the time point of highest value for each treatment. **(B)** Activation of *BcatrB* promoter at early time points shown in **(A)**. Asterisks indicate a significant difference from the control as assessed by two-tailed Student’s *t*-test, **P < 0.01, *P < 0.05.

To investigate the activation profile of the *BcatrB* promoter in response to different phytoalexins, we produced a P_*BcatrB* : *Luc* transformant of *B. cinerea* which expresses a luciferase gene under the control of the 2 kb long promoter region of *BcatrB*. Activation of the *BcatrB* promoter was detected as luciferase-mediated chemiluminescence within 10 min after 100 µM rishitin treatment. Activation of the promoter reached its peak within 1 h and quickly declined (**Figure 7**). Kuroyanagi et al. (2022) reported that rishitin is completely metabolized into oxidized forms within 6 h after treatment initiation.

Activation of the *BcatrB* promoter was also tested with brassinin and glyceollin. Treatment of 50 µM brassinin or glyceollin induced transient activation of the *BcatrB* promoter as in the case of rishitin treatment. However, the induction peak of *BcatrB* expression under glyceollin treatment occurred significantly later than those induced by rishitin and brassinin (**Figure 7A**). Likewise, activation of the *BcatrB* promoter by the brassinin treatment occurred within 15 min, whereas weak activation was detected 30 min after glyceollin treatment (**Figure 7B**). These results suggest that the induction of *BcatrB* expression by rishitin/brassinin and glyceollin might be activated by different regulatory mechanisms.

## Discussion

### Phytoalexins produced from different plant families induce differential gene expression of a suite of pathogenicity and virulence factors

Necrotrophs and generalist fungal pathogens induce cell death and lead to global tissue damage, which can lead to an increased release of plant chemical defense materials. Transcriptional reprogramming aids generalists in fine-tuning their molecular toolkits to reduce the extent of damage and mount an effective defense response by modulating antifungal metabolites such as phytoalexins (Newman and Derbyshire, 2020; Westrick et al., 2021). On top of this, there is also tight regulation in fungal pathogens in response to phytoalexins leading to continual flux and temporal variation of transcripts, compounded by the interaction of generalist pathogens across plant hosts (Westrick et al., 2019; Reboledo et al., 2020; Chen et al., 2022).

In this study, *BcatrB*, together with *BcatrD* and *Bmr3* were significantly upregulated upon rishitin treatment. These genes are likely responsible for the efflux of rishitin to enhance the tolerance of *B. cinerea*. Moreover, capsidiol and resveratrol mobilize distinct transcriptional programming as compared to that by rishitin. These findings suggest that the effective transporters differ depending on the phytoalexins, and *B. cinerea* could be inducing expression of the appropriate transporter genes by recognizing different phytoalexins. ABC transporter BcatrB has been reported to export structurally unrelated phytoalexins such as resveratrol, camalexin (Vermeulen et al., 2001; Stefanato et al., 2009), as well as the fungicide fenpiclonil (Schoonbeek et al., 2001). Likewise, a number of studies link the transcriptional activation of efflux ABC transporter genes such as *BcatrB* and *BcatrD* to induction by phytoalexins and fungicides (Hayashi et al., 2001; Perlin et al., 2014; Seifbarghi et al., 2017). On the other hand, upregulation of *Bmr1* and *Bmr3* has been reported in response to several fungicides, a phytoalexin (resveratrol) and other toxic metabolites (Makizumi et al., 2002). Similar upregulation of such transporters has been reported in other fungal necrotrophs. Treatment of indolic phytoalexins and glucosinolate breakdown products to *Alternaria brassicicola* has also resulted in transcriptional activation of drug efflux transporters (Sellam et al., 2007). Transporter-encoding genes were also induced among *B. cinerea*-infected strawberry fruits (Xiong et al., 2018). These findings offer proof that *B. cinerea*, along with other necrotrophs, could induce the expression of the appropriate transporter genes by recognizing different phytoalexins.

Previous studies have also elaborated on the role of *B. cinerea*’s toolkit of cell wall degrading enzymes (CWDEs) as virulence factors. Knockout of a polygalacturonase gene *Bcpg1* had no effect on primary infection but caused significant decrease in secondary infection (development of the lesion) (ten Have et al., 1998). BcXyn11a, encoding an endo-β-1,4-xylanase for the degradation of hemicellulose, is shown to be required for full virulence of *B. cinerea* (Brito et al., 2006). In addition, production of toxic secondary metabolites such as botcinins (polyketide synthesis genes) also facilitate fungal pathogens in subduing plant defenses (Moraga et al., 2021; Bi et al., 2022; Leisen et al., 2022). Given that these genes involved in the spread of disease symptoms are also induced by rishitin in *B. cinerea*, implies that phytoalexins produced by plants as resistance factors are used by *B. cinerea* as cues to promote virulence. Modulations in the transcriptomes of *B. cinerea* and other necrotrophs enable these pathogens to counter diverse plant defense strategies by activating virulence factors involved in xenobiotic metabolism, efflux of multiple toxic substrates, enhanced necrotrophy by means of CWDEs, and production of fungal phytotoxic metabolites.

### Evolution of versatile fungal cytochrome p450s in detoxifying phytoalexins

Degrading toxic plant metabolites is critical for the pathogenicity of *B. cinerea* (Rodríguez-Bonilla et al., 2011; Hahn et al., 2014; Valero-Jiménez et al., 2019; Leisen et al., 2022). It is not surprising then to find considerable variation among fungal pathogens in terms of breaking down phytoalexins into less toxic forms. In some cases, detoxification of phytoalexins may only involve the action of a single enzyme, such as in the case of capsidiol (Kuroyanagi et al., 2022) and pisatin (Matthews and Van Etten, 1983). On the other hand, a series of enzymatic reactions catalyze the conversion of cruciferous phytoalexins such as brassinin and camalexin into their less toxic forms. This multi-enzyme catalysis of detoxification reactions was shown across a range of fungal pathogens including necrotrophs such as *B. cinerea* and *Sclerotinia sclerotiorum* (Pedras et al., 2011; Pedras and Abdoli, 2017; Kuroyanagi et al., 2022). In a similar circumstance, a brassinin detoxification factor, Bdtf1 together with 10 other putative enzymes were reported to be important in the chemical modification of brassinin by *Alternaria brassicicola* (Cho et al., 2014). Differential gene expression of cytochrome P450 genes have also aided *S. sclerotiorum* in navigating across phytoalexin substrates from phylogenetically distant dicot species (Kusch et al., 2022). Stilbene-phytoalexins are detoxified by a single peroxidase gene (*POX*) to yield three oxidation products (Rodríguez-Bonilla et al., 2011). Despite capsidiol and rishitin having similar structural profiles, *B. cinerea* employs different mechanisms for the detoxification process, both in terms of sequential steps in detoxification and number of oxidized products (Kuroyanagi et al., 2022). In this study, oxidation of rishitin was catalyzed by two oxidoreductases (Bcin16g01490 and Bcin08g04910). However, these enzymes were still unable to account for three other metabolites present in the wild type. Previous reports have provided us with alternative scenarios on possible enzymatic transformation processes.

Studying events that led to the diversity of detoxification processes in fungal systems may also provide valuable clues towards mining possible enzyme pathways. Expansion of cytochrome P450 genes catering to phytoalexin substrates likely resulted from both convergent and divergent evolution events (Jawallapersand et al., 2014; Shang et al., 2016). At the division level, cytochrome P450 genes have undergone several divergences in the course of fungal evolution, with highly conserved structural motifs but very low sequence similarity (Chen et al., 2014). Moreover, horizontal gene transfer and gene duplication also drove cytochrome P450 diversification (Syed et al., 2014). Fungal cytochrome P450s have complex evolutionary histories at all taxonomic levels that enable expansion of roles towards detoxifying toxic plant metabolites such as phytoalexins. Understanding these complexities is crucial in crafting alternative strategies to mitigate the damage caused by *B. cinerea* across several crop species.

### Efflux and enzymatic detoxification of phytoalexins act in tandem across several filamentous fungi

Phytoalexins induce damage to cell ultrastructure as well as conidial germination of *B. cinerea* (Adrian and Jeandet, 2012). While BcatrB has been shown to be an important efflux transporter for a variety of anti-microbial chemicals, other transporters have also been reported to be involved in resistance of *B. cinerea* to toxins. Deletion of MFS transporters in *B. cinerea*, *Bcmfsg* and *Bcmfs1*, resulted in the increased sensitivity to natural toxic compounds produced in plants (Hayashi et al., 2002; Vela-Corcía et al., 2019). These previous reports clearly demonstrate the use of multiple ABC and MFS transporters in reducing damage to *B. cinerea* by removal of these chemicals, natural or artificial, by efflux transport mechanisms.

In this study, it was shown that *B. cinerea* employs two important processes such as efflux and enzymatic detoxification to tolerate phytoalexins and, hence enhancing the virulence. In particular, functional analysis revealed that *ΔbcatrB* had reduced pathogenicity on red clover leaf and lesser sporulation rate in tomato fruits. Tolerance to phytoalexins has also been documented for other fungal pathosystems as well. *Gibberella pulicaris* ABC transporter Gpabc1, sharing high homology with *Magnaporthe oryzae* ABC1, was also shown to be a crucial virulence factor for tolerating rishitin and conferring virulence in potato (Fleißner et al., 2002). Moreover, *Nectria haematococca* also utilized the two-pronged approach to detoxifying pisatin through the ABC transporter Nhabc1 and a cytochrome P450. Moreover, Nhabc1 is also phylogenetically related to both Gpabc1 and MoABC1 (Coleman et al., 2011). Interestingly, the proponents suggested that detoxification occurred in a step-wise manner, with the energy for transporting pisatin being used as springboard to propel its enzymatic transformation. This coordinated approach was also shown in the xenobiotic metabolism in *Sclerotinia homeocarpa.* In a multi-drug resistant field strain of *Sclerotinia homeocarpa*, various stages of detoxification are coordinated by a mutated transcription factor ShXDR1. Heterologous expression of mutated (dominant active) ShXDR1 in *B. cinerea* also increased expression of a cytochrome P450 (*BcCYP65*) and a transporter (*BcatrD*) leading to tolerance to multiple fungicides (Sang et al., 2018). These findings imply that this tandem strategy is highly conserved among filamentous fungi and may be a viable and more effective biocontrol approach to destructive fungal pathogens.

Differential expression of *BcatrB* was prompted by a localized production of rishitin and resveratrol in fruits of tomato and grape, respectively. Similarly, in this study, there was also tissue-specific expression of GFP under the control of the *BcatrB* promoter. Infection cushions have been shown to be formed alternatively with appressoria during less favorable conditions, to facilitate infection by penetration of the host tissue and upscaling virulence factors (Choquer et al., 2021; Bi et al., 2022).

Previous analysis revealed that some fungicide-resistant *B. cinerea* isolated from fields express high levels of *BcatrB*. A common mutation in these isolates was found in the coding sequence of the Zn(II)_2_Cys_6_-type transcription factor Mmr1 (Kretschmer et al., 2009), suggesting that adapting the transcriptional regulation of gene(s) for drug resistance transporters, like *BcatrB*, is an important process in the evolution of gray mold fungi to become pleiotropic pathogens. *BcatrB* homologues, and proximal genes/orthologues, are widely conserved in the *Botrytis* genus as well as closely related taxa (data not shown). However, there is variability in host ranges, with most representatives having narrow or limited host ranges. Moreover, GFP expression under the BcatrB promoter was limited to plants belonging to Family Solanaceae, Brassicaceae and Fabaceae. Thus, it will be interesting to examine whether *Botrytis* sp. with a narrow host range can induce the expression of *BcatrB* orthologs in response to phytoalexins from the nightshade, crucifer and bean families (rishitin, brassinin, and glyceollin/medicarpin). A detailed analysis of the *BcatrB* promoter may provide clues as to whether there are multiple *cis* elements involved in fine-tuning the expression of *BcatrB* across phytoalexin treatments.

This current study details how *B. cinerea* employs a two-pronged approach towards detoxifying rishitin. Using transcriptomic data, we discovered the utilization of an ABC transporter BcatrB and two oxidoreductases in metabolizing rishitin by efflux or oxidation, respectively. Genes for cell wall degrading enzymes as well as botcinin production were also upregulated by rishitin treatment. This provided proof of the oxidation of rishitin by two oxidoreductases. *ΔbcatrB* strains also exhibited increased sensitivity to rishitin, while having reduced pathogenicity in red clover and tomato fruits. In addition, *B. cinerea* expressing GFP under the control of the *BcatrB* promoter was activated upon infection of leaves of plants belonging to Solanaceae, Brassicaceae and Fabaceae plants. Likewise, phytoalexins that were produced from these plants also induced the activation of the *BcatrB* promoter, indicating *BcatrB* expression is induced by *B. cinerea* recognizing indole and pterocarpan phytoalexins. Under the luciferase reporter assay, *BcatrB* expression is presumed to have different *cis*-regulatory elements for the efflux of rishitin, brassinin and glyceollin. Further research into the regulation of these promoter elements may be crucial towards further understanding the flexibility of *B. cinerea* in tolerating a wide diversity of phytoalexins.

Overall, the diversity of strategies employed by fungal pathogens to overcome plant defenses highlights the complex and dynamic nature of the interactions between plants and their pathogens. Understanding these interactions and the mechanisms underlying them is essential for developing effective strategies for managing fungal diseases in agricultural and natural ecosystems in the future.

## Data availability statement

The datasets presented in this study can be found in online repositories. The names of the repository/repositories and accession number(s) can be found below: https://www.ncbi.nlm.nih.gov/genbank/, DRA013980.

## Author contributions

DT designed the research. AB, TK, AA, KI, MO, and DT conducted the experiments. AB, MC, AT, and DT analyzed data. IS, SC, MO, and DT supervised the experiments. AB, MC, IS, SC, and DT contributed to the discussion and interpretation of the results. AB and DT wrote the manuscript. AB, MC and DT edited the manuscript. All authors contributed to the article and approved the submitted version.
