# Supplementary material for: Botrytis cinerea tolerates phytoalexins produced by Solanaceae and Fabaceae plants through an efflux transporter BcatrB and metabolizing enzymes
Source: Front Plant Sci. 2023 Jun 2;14:1177060. doi: 10.3389/fpls.2023.1177060 (PMC10273015; doi:10.3389/fpls.2023.1177060)
Supplement: Supplementary file 1 [file DataSheet_1.docx]

**Supplementary Information for**

*Botrytis cinerea* tolerates phytoalexins produced by Solanaceae and Fabaceae plants through an efflux transporter BcatrB and metabolizing enzymes

**Authors:**

Abriel Salaria Bulasag, Maurizio Camagna, Teruhiko Kuroyanagi, Akira Ashida, Kento Ito, Aiko Tanaka, Ikuo Sato, Sotaro Chiba, Makoto Ojika and Daigo Takemoto

**SUPPLEMENTARY FIGURE S1 |** Metabolization of rishitin by *Epichloë festucae* transformants expressing rishitin-induced *B. cinerea* gene Bcin16g01490 encoding cytochrome P450. Mycelial blocks (approx. 1 mm^3^) of *E. festucae* transformant expressing Bcin16g01490 were incubated in 50 µl of 100 µM rishitin for 0 h or 10 days and rishitin and oxidized rishitin were detected by LC/MS.

**SUPPLEMENTARY FIGURE S2 |** Targeted gene replacement of the *B. cinerea BcatrB* locus.

**(A)** Physical map of the *BcatrB* wild-type (WT) genomic region, linear insert of *BcatrB* replacement construct pNPP214 and complementation construct pNPP215, showing restriction enzyme sites for *Spe*I (S) and *Pst*I (P). The mutated genomic locus of the *BcatrB* deletion mutant (*ΔbcatrB*) is depicted to show homologous recombination of the *hph* cassette. Primers used for the construction of deletion and complementation vectors and screening for the replacement event are indicated by arrows. **(B)** Confirmation of gene disruption in isolated *ΔbcatrB* strains by PCR. Genomic DNA from *B. cinerea* WT and *ΔbcatrB* strains were used for PCR with indicated primers.

**SUPPLEMENTARY FIGURE S3 |** Pathogenicity assay of *B. cinerea* wild type (WT) and complemented strain *ΔbcatrB*-14-C1 in tomato and red clover. **(A)** Tomato fruits (cut in half) were inoculated with mycelia plug (approx. 5 x 5 mm) of *B. cinerea* wild type (WT) or *ΔbcatrB-*14-C1 strain and produced conidia were counted 7 days after the inoculation. Data are mean ± SE (n = 8). **(B)** Leaves of red clover were inoculated with mycelia plug (approx. 5 x 5 mm) of *B. cinerea* WT or *ΔbcatrB*-14-C1 and lesion diameter was measured 3 days after the inoculation. Data are mean ± SE (n = 9). N. S. indicate no significant difference from WT as assessed by two-tailed Student’s *t*-test.


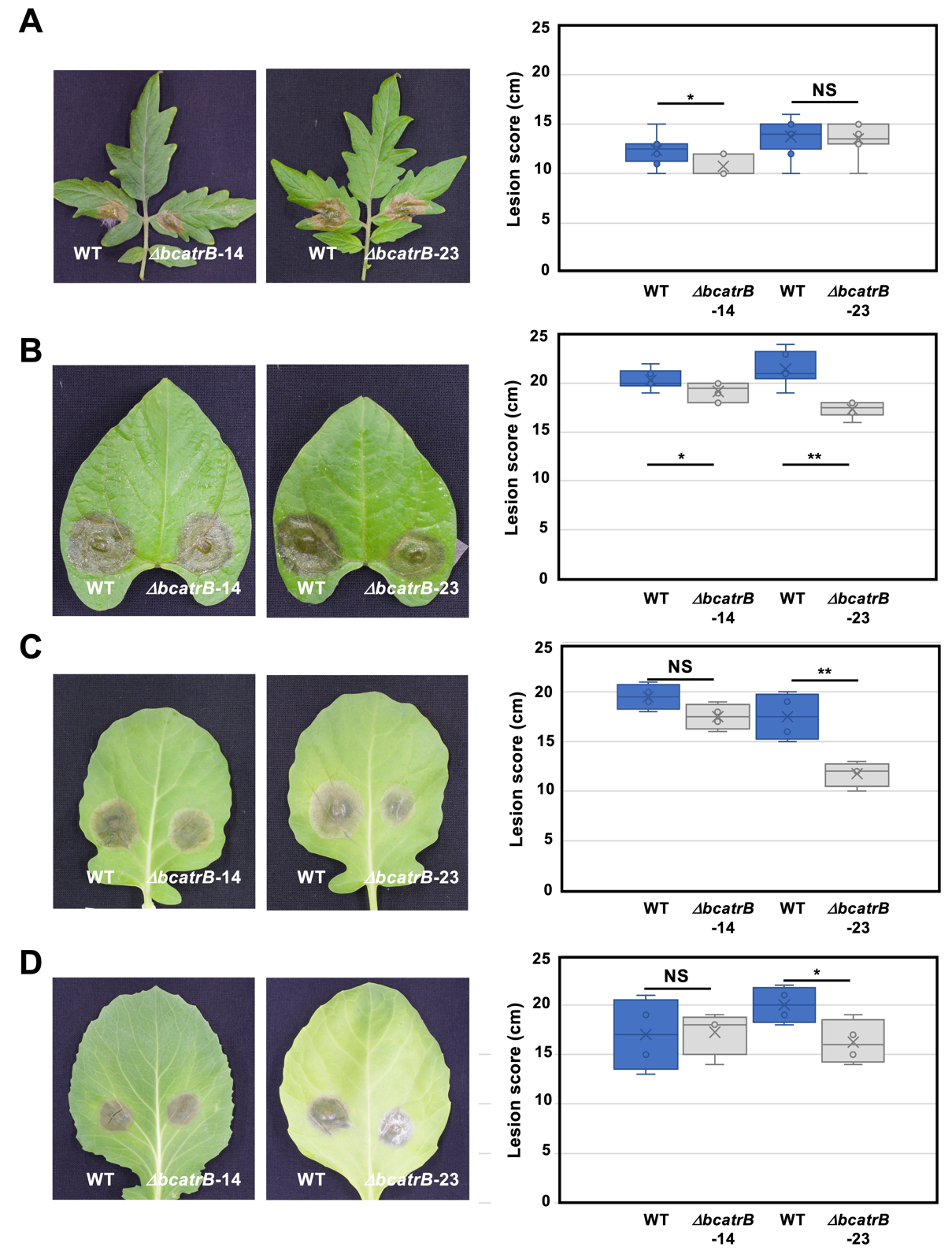


**SUPPLEMENTARY FIGURE 4** | Effect of *BcatrB* KO on pathogenicity of *Botrytis cinerea* in tomato, green bean, broccoli, and cabbage. Leaves of tomato (A), green bean (B), broccoli (C), or cabbage (D) were inoculated with mycelia plug (approx. 5 x 5 mm) of *B. cinerea* wild type (WT), *ΔbcatrB*-14 or *ΔbcatrB*-23 and lesion diameter was measured 2 days after the inoculation. Data are mean ± SE (n = 6). Significant difference from WT was assessed by two-tailed Student’s t-test. N. S., not significant.
